# Cysteine Alkylation in Enzymes and Transcription Factors: A Therapeutic Strategy for Cancer

**DOI:** 10.3390/cancers17111876

**Published:** 2025-06-03

**Authors:** Celia María Curieses Andrés, Fernando Lobo, José Manuel Pérez de la Lastra, Elena Bustamante Munguira, Celia Andrés Juan, Eduardo Pérez-Lebeña

**Affiliations:** 1 Hospital Clínico Universitario de Valladolid, Avenida de Ramón y Cajal, 3, 47003 Valladolid, Spain; cmcuriesesa@saludcastillayleon.es (C.M.C.A.); ebustamante@saludcastillayleon.es (E.B.M.); 2 Institute of Natural Products and Agrobiology, CSIC-Spanish Research Council, Avda. Astrofísico Fco. Sánchez, 3, 38206 La Laguna, Spain; fernando.lobo@csic.es; 3 Cinquima Institute and Department of Organic Chemistry, Faculty of Sciences, Valladolid University, Paseo de Belén, 7, 47011 Valladolid, Spain; celia.andres.juan@uva.es; 4 Sistemas de Biotecnología y Recursos Naturales, 47625 Valladolid, Spain; info@glize.eu

**Keywords:** transcriptional and enzyme regulation, binding at cysteine residues, natural alkylating agents, cancer cells

## Abstract

Metabolic enzymes and cancer-driving transcriptions factors are often overexpressed in neoplastic cells, and their exposed cysteine residues are amenable to chemical modification. This review explores cysteine alkylation as a cancer treatment strategy, focusing on Michael acceptors like curcumin and helenalin, which interact with transcription factors NF-κB, STAT3 and HIF-1α. Molecular docking studies using AutoDockFR revealed distinct binding affinities: curcumin showed strong interactions with STAT3 and NF-κB, while helenalin exhibited high affinity for STAT3 and HIF-1α. Synthetic compounds like STAT3-IN-1 and CDDO-Me demonstrated superior binding in most targets, except for CDDO-Me with HIF-1α, suggesting unique structural incompatibilities. Natural products such as zerumbone and umbelliferone displayed moderate activity, while palbociclib highlighted synthetic-drug advantages. These results underscore the importance of ligand−receptor structural complementarity, particularly for HIF-1α’s confined binding site, where helenalin’s terminal Michael acceptor system proved optimal. The findings advocate for integrating computational and experimental approaches to develop cysteine-targeted therapies, balancing synthetic precision with natural product versatility for context-dependent cancer treatment strategies.

## Simple Summary

This study explores a new cancer treatment strategy that targets specific cysteine residues in enzymes and transcription factors commonly overexpressed in cancer cells. It focuses on the use of natural compounds -like curcumin, cinnamaldehyde, zerumbone, helenalin and umbelliferone- that can form covalent bonds through a chemical reaction called Michael addition. Using computational docking tools, the study compares how well these natural products and standard cancer drugs bind to key proteins such as NF-κB, STAT3 and HIF-1α. Results suggest that some natural compounds, especially curcumin, show promising binding abilities, highlighting their potential as low-toxicity therapeutic options. This approach combines literature review and computer simulations to support future drug discovery.

## 1. Introduction

Neoplastic cells exhibit distinct metabolic alterations compared to normal cells, and these alterations are driven by changes in enzyme activity and transcriptional regulation, which contribute to the uncontrolled growth and proliferation characteristic of cancer. Understanding the metabolic alterations and regulatory mechanisms has led to the development of new therapeutic strategies. Targeting key enzymes or transcription factors (TFs) involved in cancer metabolism can offer novel approaches to cancer treatment [1].

Residues of cysteine (Cys) and selenocysteine (Sec), with their thiol (-SH) and selenol (-SeH) groups, are vital for the structure and function of enzymes and TFs. Due to their high reactivity, they act as molecular switches in response to cellular stimuli. Their sensitivity to oxidation allows them to form bonds with electrophilic compounds, altering protein structure and function, making them ideal sensors for changes in the cellular redox state [2]. Cys residues can undergo various post-translational modifications (PTMs), such as S-nitrosylation, electrophilic attack or oxidation by reactive oxygen species (ROS) [3]. These modifications can alter the activity, localisation, interaction with other proteins and TFs or may alter its affinity for DNA, thereby modifying the TF’s ability to bind to its response elements in the genome [4].

**Figure 1:**
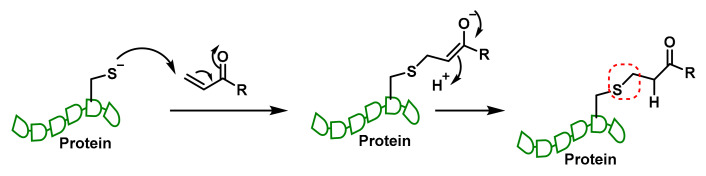
The Michael reaction with the thiolate group of Cys in proteins and enzymes.

The Michael reaction, discovered by Arthur Michael in the late 1880s, involves a nucleophilic compound reacting with an electrophile to form C-C bonds efficiently. In Michael additions, C–Nu bonds are typically formed when a nucleophile (Nu^−^) adds to an α,β-unsaturated carbonyl compound (a Michael acceptor). The result is a new carbon–nucleophile bond at the β-carbon of the acceptor [5]. This process typically involves adding an enolate to an activated alkene, such as an α,β-unsaturated ketone. The reaction is a type of conjugate addition and is also used to form S-C bonds [6] (Figure 1).

Thiolates typically act as nucleophiles in Michael addition reactions; therefore, the nucleophilicity and pKa of the reacting cysteine residue significantly influence its reactivity. Thiolates (RS^−^) are deprotonated thiols (RSH) and are much stronger nucleophiles than the protonated form. The pKa of the cysteine side chain determines how readily it forms the thiolate at physiological pH. A lower pKa means the thiol group is more deprotonated (i.e., more in the thiolate form) at physiological pH, thus increasing nucleophilicity and reactivity in a Michael addition [7].

Michael acceptor (MA) molecules can form covalent bonds with nucleophilic residues such as the thiol group on Cys residues and thus modulate protein pathways, playing a key role in the regulation of proteins and TFs. These molecules are candidates for the treatment of diseases such as inflammation, cancer and oxidative stress (OS) [8].

This reaction can lead to the formation of a covalent bond between the Michael acceptor and the biomolecule. This modification can alter the structure and function of the biomolecule, leading to a variety of physiological effects. Many natural compounds contain MA groups in their structures or derivatives that make them biologically active and can react with nucleophilic residues of proteins, thus offering important therapeutic effects with minimal toxicity for various diseases [9].

Conversely, a retro-Michael addition is the reverse of a Michael addition, in which a nucleophile that was previously added to an α,β-unsaturated carbonyl compound is removed, regenerating the original Michael acceptor. In a Michael addition the nucleophile (e.g., -S- thiolate) is added to the β-carbon of an α,β-unsaturated carbonyl (such as acrolein). In a retro-Michael addition, the bond between the nucleophile and the β-carbon is broken, releasing the nucleophile and restoring the double bond. The reaction is reversible when the adduct is not strongly stabilised or the nucleophile is weakly bound. The intermediate (Michael adduct) may be unstable if there is a low concentration of electrophile, the equilibrium is shifted backwards or if the adduct is not stabilised by additional hydrogen or steric bonds. In some drug designs or biological systems, this reversibility is exploited to allow controlled reactivity or detoxification, while in others irreversibility is preferred (e.g., to permanently inactivate TrxR in cancer cells).

Both Tyrosine Kinases (TKs) and Cyclin-Dependent Kinases (CDKs) possess reactive cysteines in their active sites or in nearby regions that can be alkylated by Michael acceptors. TKs contain nucleophilic cysteines in strategic positions (e.g., in the ATP-binding region or near the catalytic pocket). These cysteines can react with drugs containing Michael acceptors, such as irreversible covalent inhibitors (e.g., afatinib and ibrutinib, which act on EGFR and BTK, respectively). However, this field is very broad and requires a comparative analysis which is outside of the scope of the present review.

This study presents a review and a proposal discussing the potential application of computational methods to predict chemical interactions with proteins overexpressed in cancer, with a particular focus on the alkylation of cysteine residues. The field of computational prediction of protein−ligand interactions is rapidly advancing in drug discovery and cancer research. In the molecular representations of the different compounds discussed, the structure of the Michael acceptor is highlighted in red.

## 2. Enzyme and Transcriptional Regulation in Neoplastic Cells

In this chapter we review a key enzyme (Thioredoxin Reductase) and up to five TFs that are overexpressed in neoplastic cells and have a major impact on the hallmarks of cancer. Two common features of this enzyme and the TFs studied are their overexpression in neoplastic cells and the presence of nucleophilic Cys residues that play a key role in metabolic functioning and are therefore susceptible to reaction with electrophilic compounds (such as MAs).

### 2.1. Thioredoxin Reductase (TrxR) and Its Role in Cancer Cells

Thioredoxin reductase (TrxR) is an enzyme that plays a key role in cellular function by maintaining redox homeostasis, protecting cells from OS. This enzyme is essential for reducing thioredoxin (Trx), which in turn reduces other proteins by Cys thiol−disulphide exchange. TrxR plays a crucial role in cancer cells by maintaining redox balance and protecting them from OS [10]:

- TrxR and Trx are overexpressed in many aggressive tumours, managing the increased reactive oxygen species (ROS) levels due to their high metabolic rate [11].
- The system of TrxR, Trx and NADPH reduces oxidised proteins and maintains cellular redox homeostasis [12].
- By reducing ROS, TrxR helps cancer cells survive, proliferate, enhance growth tumours and optimise nutrient and oxygen supply [13].
- Reduced Trx can inhibit apoptosis by binding to apoptosis signalling kinase-1 (ASK-1). In contrast, oxidised Trx loses this ability, highlighting the importance of Trx’s redox state in regulating apoptotic pathways [14].
- Due to its role in protecting cancer cells, TrxR is a potential target for cancer therapy. Inhibiting TrxR can disrupt redox balance in cancer cells, making them more susceptible to oxidative damage and apoptosis [15].

**Figure 2:**
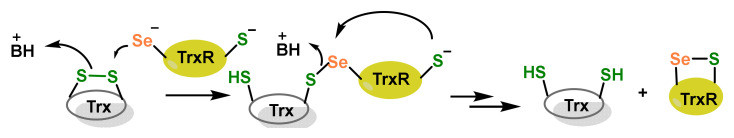
Catalytic activity of Sec in TrxR. BH^+^ represents the acid conjugate of the neutral base B. BH^+^ is the proton donor in the reaction.

Sec, known as amino acid 21, is a unique selenium-containing amino acid, crucial for the catalytic activity of TrxR due to its high catalytic efficiency, resistance to oxidation and superior electrophilicity. Sec is a better nucleophile than Cys, enhancing TrxR’s efficiency, offering greater resistance to irreversible oxidation under OS conditions and accepting electrons more effectively, making it more efficient in redox reactions [16]. The pKa value of Cys and Sec are 8.3 and 5.2, respectively, implying that at physiological pH Sec is negatively charged as selenolate (Figure 2). Enzymes containing Sec in the active site are catalytically more efficient than Cys-containing counterparts [17].

TrxR contains a redox-active centre with a CysSec moiety, an essential sequence for the electron transfer to Trx, which in turn reduces other proteins. Alkylation on an active centre can significantly affect their function and disrupt their catalytic mechanism [12]. In TrxR, the Sec residue is located at the penultimate position in the C-terminal active site [18] and is essential for the enzyme’s catalytic activity, as it plays a critical role in the reduction of Trx and other substrates [19].

**Figure 3:**
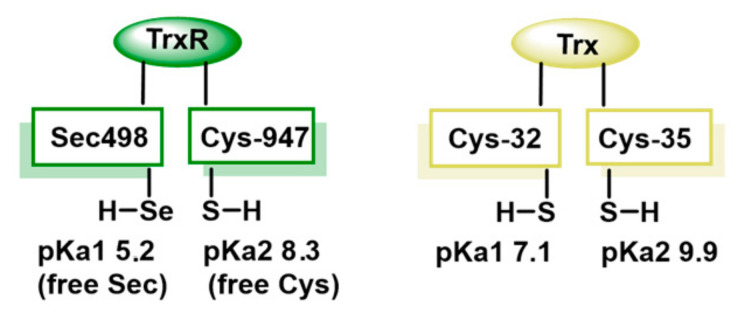
Comparison of the thiol pKa values for Trx and TrxR.

At physiological pH, both the thiol and the selenol moieties of TrxR behave as strong nucleophiles [20,21,22] (Figure 3). TrxR contains the catalytic oxidation site of Cys497-Sec498 and the catalytic reduction site of Cys59 and Cys64. Electrons are transferred from NADPH to these Cys, then to the Cys497-Sec498 pair at the C-terminal active site and finally to the Trx substrate, to exert the redox regulatory function [12]. Therefore, it is of great interest to study whether there is a structure that can react with the Se-H bond in Sec498 and the S-H bond in Cys497 to inhibit their catalytic activity [23,24,25,26,27,28].

Acrolein is typically considered a harmful and toxic compound. It is a reactive α,β-unsaturated aldehyde that can damage DNA, proteins and lipids and is a known environmental pollutant found in cigarette smoke and industrial emissions and as a byproduct of lipid peroxidation. It is often shown in biochemical contexts as a model electrophile because of its high reactivity towards nucleophilic groups on Cys or Sec residues in proteins. While acrolein itself is too toxic and non-specific to be used directly as a drug, its reactivity profile has inspired the design of selective inhibitors that can exploit the TrxR vulnerability selectively in cancer cells [29].

**Figure 4:**
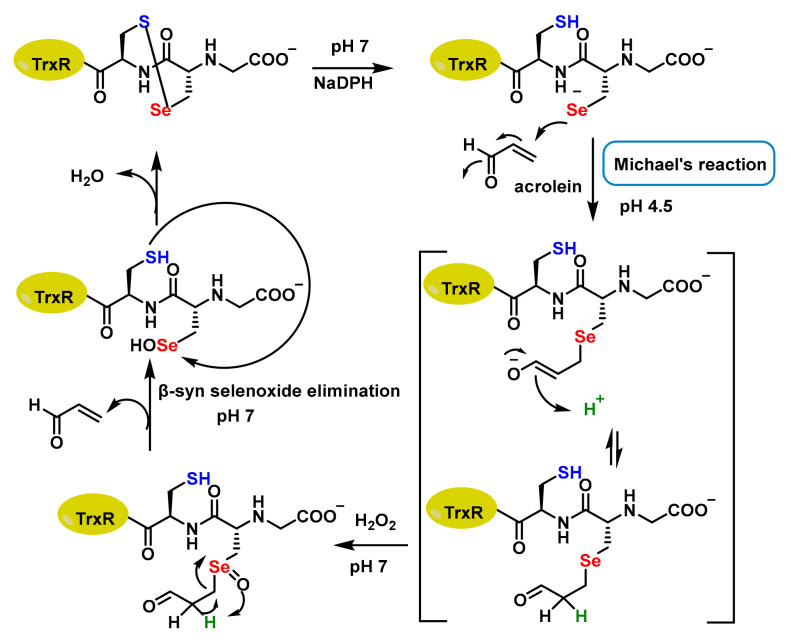
Proposed mechanism of Sec alkylation by acrolein.

Acrolein interacts with TrxR by alkylating its Sec residue, but this can be reversed through β-syn selenoxide elimination. During this process, H_2_O_2_ oxidises the selenide to selenoxide, which abstracts the β-proton near the selenium atom. This β-selenoxide elimination restores TrxR to its oxidised selenosulphide state, renewing partially the TrxR activity. The quick elimination of the β-s selenoxide enables repair of the selenoenzyme, thanks to the C-Se bond’s lability [30] (Figure 4).

Emma Mari et al., 2020, demonstrated that acrolein-inactivated Sec498 could recover 25% and 30% activity, respectively, when incubated with 2 mM H_2_O_2_ and 5 mM imidazole. In contrast, Cys497 did not recover activity under the same conditions [31]. Similarly, TrxR is irreversibly modified by curcumin, and Jianguo Fang et al., 2005, using mass spectrometry and transfer analysis, demonstrated that this inhibition was caused by alkylation of both residues at the catalytically active site (Cys497 and Sec498) of the enzyme [32].

TrxR alkylation can lead to increased OS in cancer cells, potentially making them more susceptible to treatment. TrxR and Trx are often overexpressed in many aggressive tumours, making them more dependent on the Trx system than normal cells [33]. The selective inhibition of TrxR could enhance the effectiveness of cancer treatments by making cancer cells more vulnerable to oxidative damage [34].

### 2.2. Exportin-1 (CRM1/XPO1) and Its Role in Cancer Cells

Exportin-1 (CRM1/XPO1) is a crucial nuclear export protein, because it transports a wide variety of proteins, including regulatory factors, from the nucleus to the cytoplasm. Because of its role, XPO1 is considered a potential anticancer target as it is often overexpressed and plays an important role in tumourigenesis and metastasis [35].

XPO1 exports oncoproteins, such as c-Myc, cyclin D1 and Bcl-2, from the nucleus to the cytoplasm. These proteins promote cell proliferation, survival and angiogenesis, contributing to tumour growth. XPO1 promotes the expression of genes involved in the epithelial−mesenchymal transition (EMT), a process that allows cancer cells to acquire migratory and invasive properties, inducing metastasis [36].

**Figure 5:**
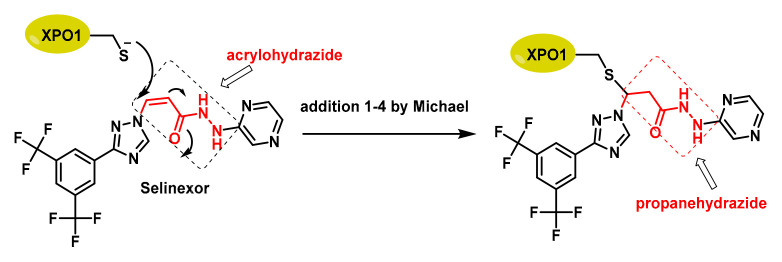
The acrylohydrazide (red) of Selinexor acts as a Michael acceptor, facing the Cys528 nucleophile of XPO-1. A propanehydrazide derivative (red) is formed and appears inside the compound box on the right.

Several XPO1 inhibitors developed are currently under clinical trials. These inhibitors block the function of XPO1, leading to the accumulation of tumour suppressor proteins and miRNAs in the nucleus and inhibiting the export of oncoproteins. Selective nuclear export inhibitors (SINEs) compounds have shown broad-spectrum anticancer activity by inhibiting XPO1-mediated nuclear export. This inhibition can disrupt the normal function of cancer cells, making them more susceptible to treatment. One of the most well-known SINE compounds is Selinexor (KPT-330) (Figure 5), for both solid tumours and haematological malignancies. Selinexor has been approved for the treatment of relapsed/refractory multiple myeloma and diffuse large B-cell lymphoma [37].

In XPO1, the most important Cys residue for its function is Cys528, which is located in the cargo-binding pocket of XPO1 and is the target of selective inhibitor of SINE compounds [38]. Alkylation of Cys528 is a key mechanism for exerting their anticancer effects, as it disrupts the interaction between XPO1 and its cargo proteins, preventing their export from the nucleus to the cytoplasm [39]. The specificity of SINE compounds for Cys528 offers a promising therapeutic approach for several types of cancer.

### 2.3. Signal Transducer and Activator of Transcription 3 (STAT3) and Its Role in Cancer Cells

In normal cells, STAT3 plays a vital role in cytokine-induced gene expression and is involved in several cellular processes: (i) it regulates cell proliferation and survival by transmitting cytokine and growth factor signals [40], (ii) it is involved in the immune system by regulating gene expression [41], (iii) STAT3 is involved in the differentiation of several cell types, including stem cells [42], and (iv) it mediates the acute phase response to inflammation by regulating protein expression [43].

In the physiological state, STAT3 is usually transient and tightly regulated, ensuring that it performs its functions without causing uncontrolled cell growth or survival [44]. Aberrant STAT3 activation is associated with inflammatory diseases and neoplastic cellular processes, such as cell growth, survival and differentiation. STAT3 promotes several hallmarks of cancer, such as tumour proliferation, metastasis, angiogenesis, immune evasion, inflammation, metabolic reprogramming and cancer growth [45]. Examples of STAT3-regulated genes include those involved in (i) cell proliferation (cyclins, CDKs and growth factors) [46], (ii) cell survival (Bcl-2 and IAPs) [47], (iii) inflammation (IL-6, TNF-α and cell adhesion molecules) [41], (iv) angiogenesis (VEGF) [48] and (v) cell differentiation [49].

The Cys259 of STAT3 are pivotal for its function, involved in the formation of the dimer and DNA binding [50]. When this residue forms disulphide bonds, they can influence the dimerization process, affecting the transcriptional activity of STAT3, as dimerization is required for DNA binding [51]. The alkylation of Cys259 is a significant area of research due to its implications in regulating STAT3 activity.

Cyanoketones, like cyanoacrylamides, can indeed act as reversible covalent inhibitors, especially via Michael-type acceptor chemistry. Both cyanoketones and cyanoacrylamides contain α,β-unsaturated carbonyl systems with an electron-withdrawing cyano group. This setup makes the β-carbon highly electrophilic and prone to Michael addition by nucleophiles like thiols or selenols from Cys or Sec residues in proteins. Cyanoacrylamides are well known as reversible covalent inhibitors, especially for targeting active-site cysteines (e.g., in kinases). Cyanoketones share a similar reactivity pattern but often form reversible adducts, depending on the pKa of the nucleophile, steric and electronic environment around the Michael acceptor and solvent and pH [52,53].

**Figure 6:**
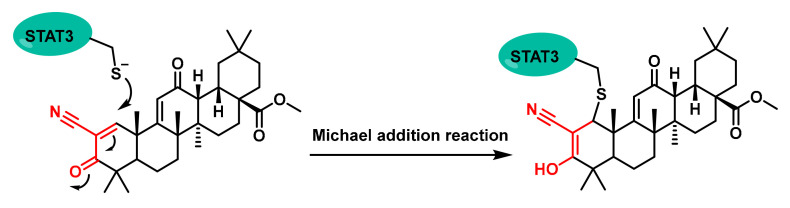
Chemical structures of a CDDO-Me-Michael addition of a reactive Cys residue.

One notable compound is CDDO-Methyl Ester (CDDO-Me) (Figure 6) [54]. This compound forms adducts with Cys259, leading to the disruption of STAT3 dimerization and subsequent inhibition of its activity [55].

This alkylation process prevents STAT3 from dimerising, which is necessary for its DNA-binding activity and subsequent gene transcription. By inhibiting this process, CDDO-Me effectively blocks the JAK1-STAT3 signalling pathway, which is overactive in various cancers [54].

**Figure 7:**
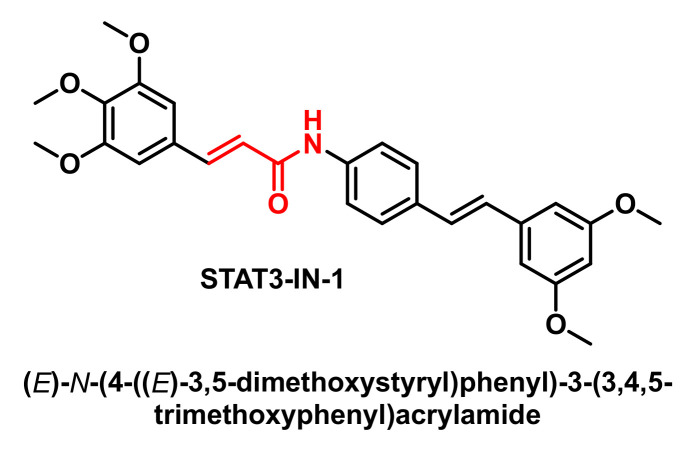
Molecular structure of STAT3-IN-1.

STAT3-IN-1 (Figure 7) is an excellent, selective and orally active inhibitor of STAT3 with IC50 values of 1.82 μM and 2.14 μM in HT29 and MDA-MB 231 cells, respectively. STAT3-IN-1 induces apoptosis of tumour cells [56,57,58].

In drug discovery and biochemical assays, “low-micromolar activity” generally refers to a compound that shows biological activity (e.g., binding to a target or inhibiting an enzyme) at concentrations in the range of 1–10 micromolar (μM). Compounds that require >10 μM or even millimolar concentrations to show activity are usually considered weak or poor binders. In contrast, low micromolar activity is significantly stronger, hence the term “excellent” in early-stage screening. A compound active at 1–5 μM is seen as more potent and promising.

### 2.4. Nuclear Factor NF-κB, IKKβ Kinase and Their Role in Cancer Cells

Nuclear Factor NF-κB is a family of TFs that play a crucial role in regulating immune responses (regulating genes involved in immune responses), inflammation (regulating the expression of cytokines, chemokines and adhesion molecules involved in inflammation) and cell survival (regulating genes that prevent apoptosis). In normal cells, NF-κB activity is tightly regulated to ensure physiological metabolism [59].

In cancer cells, NF-κB plays a significant role by promoting various processes that contribute to tumour progression and survival: (i) it drives the expression of genes that promote cell cycle progression and proliferation [60], (ii) it enhances the ability of cancer cells to invade surrounding tissues and spread to distant sites [61], (iii) it promotes the formation of new blood vessels to supply nutrients to growing tumours [62], (iv) it helps cancer cells evade the immune system by creating an immunosuppressive tumour microenvironment [63], (v) it is involved in chronic inflammation, which can contribute to tumour development and progression [64], (vi) it inhibits apoptosis and ferroptosis, promoting cell survival [65], (vii) it can either stimulate or suppress autophagy, depending on the context [66], (viii) it supports the self-renewal and survival of cancer stem cells, which are thought to drive tumour recurrence and resistance to therapy [67], and (ix) it reduces tumour cell sensitivity to chemotherapy and radiotherapy [68]. Given its central role in cancer, NF-κB is a promising therapeutic target [69]. Examples of NF-κB-regulated genes are the following:

- Cytokines and chemokines, such as IL-1β (a pro-inflammatory cytokine involved in acute and chronic inflammation), TNF-α (a potent pro-inflammatory cytokine), IL-6 (a cytokine involved in inflammation and immune response), IL-8 (a chemokine that attracts neutrophils to sites of inflammation) and MCP-1 (monocyte chemoattractant protein-1, a chemokine that attracts monocytes and macrophages) [59];
- Cell survival and proliferation genes, such as Bcl-2 (an anti-apoptotic protein that inhibits cell death), c-myc (a proto-oncogene involved in cell proliferation), cyclins and CDKs (proteins involved in cell cycle regulation) [70];
- Inhibitors of apoptosis proteins such as c-IAP1 and c-IAP2 [71] and other genes, such as ICAM-1 (intercellular adhesion molecule-1, involved in cell adhesion) [72], VCAM-1 (vascular cell adhesion molecule-1, involved in cell adhesion) [73], COX-2 (cyclooxygenase-2, an enzyme involved in inflammation and pain) [74], iNOS (inducible nitric oxide synthase, an enzyme that produces nitric oxide) [75] and MMPs (matrix metalloproteinases, involved in extracellular matrix degradation) [59].

**Figure 8:**
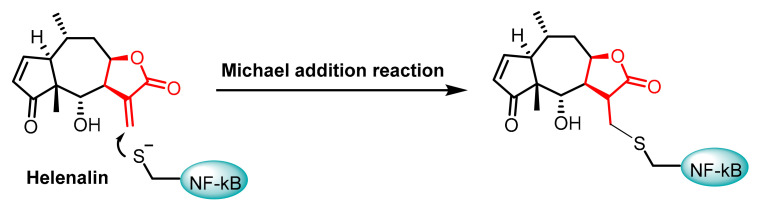
Chemical structures of the Helenalin-Michael addition of the Cys38 residue in NF-kB.

AJ García-Piñeres et al., 2001, demonstrated that sesquiterpene lactones play a crucial role in the inhibition of DNA binding by p65/NF-kB by alkylating Cys38 (Figure 8) [76]. The p65 subunit of NF-κB contains several Cys residues that are important for its function, with Cys38 being the most notable [77]. This residue is highly sensitive to the redox state, which means that changes in the redox environment can significantly impact NF-κB’s ability to function properly. In a reduced state, Cys38 enhances the DNA-binding capability of the p65 subunit, while oxidative modifications can hinder this activity [78].

The IκB kinase (IKK) complex plays a crucial role in the activation of NF-κB. The IKK complex consists of three subunits: IKKα, IKKβ and NEMO (IKKγ). While both IKKα and IKKβ are catalytically active, IKKβ is the subunit directly responsible for the phosphorylation of IκB proteins, which leads to their degradation and subsequent NF-κB activation [79]. Various stimuli, such as pro-inflammatory cytokines (like TNFα and IL-1β), bacterial lipopolysaccharide (LPS) and viral infections, can activate the IKK complex [80]. Once activated, IKKβ phosphorylates IκB proteins at specific serine residues, and this event marks IκB for ubiquitination by an E3 ubiquitin ligase complex. With IκB degraded, NF-κB is now free to translocate into the nucleus [81].

Cys179 is a specific amino acid residue located in the activation loop of IKKβ and is crucial for the proper functioning of IKKβ and its role in NF-κB activation. Cys179 is a potential target for redox regulation of IKKβ activity [82].

### 2.5. Hypoxia-Inducible Factor 1 (HIF-1) and Its Role in Cancer Cells

In normal cells, Hypoxia Inducible Factor 1 (HIF-1) plays a crucial role in regulating cellular responses to hypoxia (low oxygen levels), helping to balance oxygen homeostasis by activating genes involved in processes such as cell survival, metabolism and angiogenesis. HIF-1 ensures that cells adapt to changes in oxygen availability, promoting their survival and function under varying conditions [83].

HIF-1 activates a wide range of genes involved in physiological and pathological processes, including the following: (i) erythropoiesis (erythropoietin (EPO) that stimulates red blood cell production) [84]; (ii) angiogenesis (vascular endothelial growth factor (VEGF) that promotes blood vessel formation) [85], platelet-derived growth factor (PDGF) that stimulates vascular smooth muscle cell proliferation and migration [86]; (iii) glucose metabolism (glucose transporter 1 (GLUT1) that increases glucose uptake), hexokinase 2 (HK2) that enhances glycolysis, pyruvate dehydrogenase kinase 1 (PDK1) that inhibits pyruvate oxidation in mitochondria, promoting glycolysis [87]; (iv) cell survival (Bcl-2 family proteins that regulate apoptosis and heat shock proteins that protect cells from stress) [88]; and (v) other genes, such as inducible nitric oxide synthase (iNOS) [89] and carbonic anhydrase IX (CAIX) which regulate pH balance [90] and other glycolytic enzymes promoting glycolysis for energy production [87,91].

In cancer cells, HIF-1 plays a key role in adapting to the hypoxic conditions of the tumour microenvironment by activating genes involved in various processes such as cell survival, proliferation, angiogenesis and metastasis. Overexpression of HIF-1 in cancer cells can lead to treatment resistance and disease progression. Targeting HIF-1 has become a promising therapeutic strategy in cancer treatment [92].

**Figure 9:**
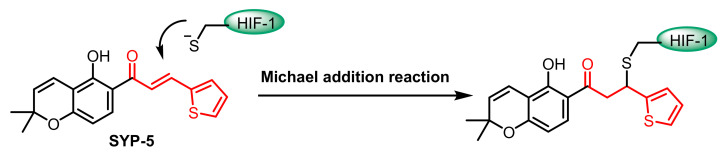
Chemical structures of the SYP-5-Michael addition of the Cys255 residue in HIF-1α.

Cys255 in HIF-1α has been identified as a new site for the development of covalent inhibitors of the protein−protein interaction of the PasB domain of HIF-1α/ARNT [93]. SYP-5 (Figure 9) is an inhibitor of HIF-1 that suppresses tumour cell migration and invasion, as well as tumour angiogenesis [94].

## 3. Synthetic and Natural Products as Alkylating Agents for Cysteine Residues

In the field of cancer treatment, numerous molecules have been developed that act as MA [9]. Some examples are the following:

- Afatinib, Neratinib, Sunitinib, Osimertinib and Ibrutinib, as Tyrosine Kinase inhibitors (TKIs). TK plays a crucial role in the signalling pathways that regulate cell division and survival, and TKIs can help control the growth of cancer cells [95].
- Palbociclib, Ribociclib, Trilaciclib and Dalpiciclib as Cyclin-Dependent Kinases (CDKs) inhibitors. CDKs play a crucial role in regulating the cell cycle by interacting with cyclins and inhibitors halt cell division and proliferation [96].
- Nitro Fatty Acids (NO_2_-FAs), which have broader biological effects and inhibit the activity of NF-κB, are bioactive lipids formed by the reaction of unsaturated fatty acids (UFAs) with reactive nitrogen species like NO and nitrite anions. NO_2_-FAs possess a nitroalkene moiety, which is a potent Michael acceptor, allowing them to undergo nucleophilic attacks on thiol groups of biologically relevant proteins [97].
- Selective Inhibitors of Nuclear Export (SINEs) as inhibitors of XPO1. Several compounds have been developed, such as Verdinexor, Selinexor and Eltanexor. By inhibiting XPO1, these drugs help keep tumour suppressor proteins inside the nucleus, which can induce cancer cell death [98].
- MA-based covalent inhibitors represent a critical class of targeted cancer therapies, particularly for hard-to-drug proteins like KRAS G12C. Sotorasib and Adagrasib are small-molecule inhibitors designed to target the KRAS G12C mutation, a common oncogenic driver in non-small cell lung cancer (NSCLC) and other cancers. This mutation results in a permanently active K-Ras protein that drives cancer cell proliferation. Sotorasib is the first approved KRAS G12C inhibitor. It binds irreversibly to the mutant KRAS G12C protein, locking it in an inactive GDP-bound state, thereby inhibiting downstream signalling pathways such as MAPK/ERK. Adagrasib is another KRAS G12C inhibitor, with similar mechanisms, developed to overcome limitations of resistance or suboptimal responses seen in some Sotorasib-treated patients. It has shown promise in both monotherapy and combination regimens.
- SHP2 (Src homology region 2-containing protein tyrosine phosphatase 2) is indeed a key cancer-related protein and a promising target for covalent inhibition, especially via Michael acceptor-based strategies. Mutations or overactivation of SHP2 is associated with leukemias, solid tumours and RASopathies. Cys459 (in the PTP domain) is the nucleophilic cysteine required for enzymatic dephosphorylation. Although directly targeting this site poses a risk of off-target effects due to high conservation among PTPs, it remains a feasible option.

In order to carry out the molecular docking study, we used the AutoDockFR algorithm, which incorporates covalent docking methods. We have selected two well-known molecules that are widely used in conventional chemotherapy, although their selectivity is higher than that of TKIs and CDKIs, namely Sunitinib and Palbociclib.

Sunitinib is an inhibitor of multiple TKRs, such as vascular endothelial growth factor receptor (VEGFR) that can be alkylated at Cys919 [99], platelet-derived growth factor receptor (PDGFR) that can be alkylated at Cys940 and Cys822 [100], stem cell factor receptor (KIT) that can be alkylated at Cys788 in the ATP-binding pocket [101] and FMS-like TK (FLT3), a cytokine receptor belonging to the receptor tyrosine kinase class III and plays a crucial role in hematopoiesis, which can be alkylated at Cys828 and is located directly preceding the DFG motif at the start of the activation loop [102]. Sunitinib is effective in the treatment of renal cell carcinoma (RCC) [103], gastrointestinal stromal tumours (GISTs) [104] and pancreatic neuroendocrine tumours (pNETs) [105,106]. Although not an approved use, it has been investigated for efficacy in NSCLC [107] and prostate cancer [108].

**Figure 10:**
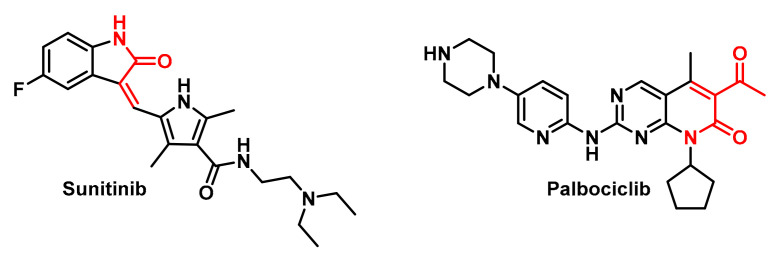
Molecular structures of Sunitinib and Palbociclib.

Palbociclib is primarily used for the treatment of metastatic or advanced hormone receptor (HR)-positive and HER2-negative breast cancer, in combination with an aromatase inhibitor as an initial treatment or with Fulvestrant in women who have received prior hormone therapy [109]. Palbociclib works by blocking the CDK4 and CDK6 proteins with IC50 values of 11 nM and 16 nM, respectively, which regulate cell growth and division. According to Emi Kishino et al., 2019, Palbociclib reduced Rb phosphorylation and cell growth in association with G1-S cell cycle blockade and the induction of cell senescence, but without increased apoptosis, and can slow the growth of cancer cells and delay cancer progression [110]. These synthetic molecular compounds are shown in Figure 10.

**Figure 11:**
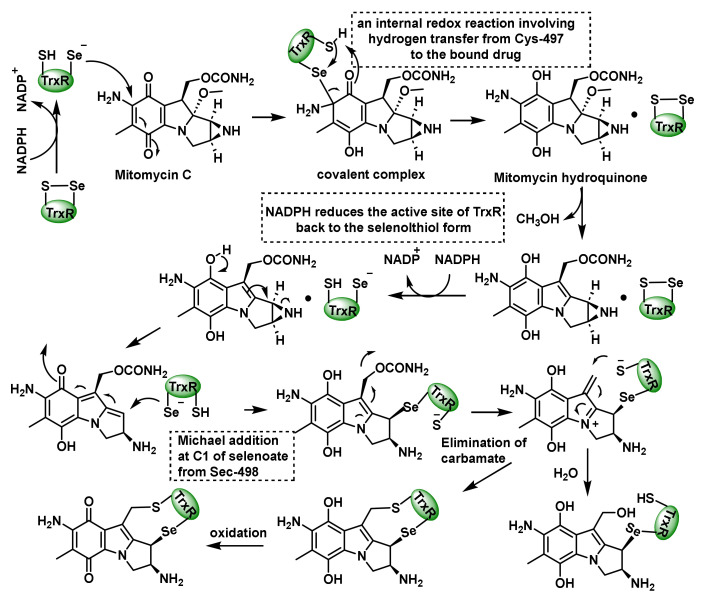
Mechanism for the mechanism-based inhibition of TrxR by MMC.

Natural products have played a significant role in the development of anticancer therapies, including alkylating agents. These compounds, derived from natural sources like plants, microorganisms and marine organisms, exhibit a wide range of biological activities, including DNA alkylation [111]. An example of natural products that perform as an alkylating agent is mitomycin C (MMC) (Figure 11).

Paz et al., 2012, proposed that MMC inactivates TrxR in cancer cell cultures [112]. The proposed mechanism starts with a selenoate conjugate addition of the Sec498 residue to the quinone ring of MMC, which undergoes an internal redox reaction involving a hydrogen transfer from Cys497 to the MMC hydroquinone, generating an active intermediate, while NADPH reduces the TrxR active site back to the seleno−thiol form [113]. The next step is the removal of carbamate and the addition of thiolate from Cys497, forming a cross linker in the active site. The last step is the oxidation of the hydroquinone ring. Alternatively, the addition of H_2_O at the second electrophilic position inactivates the enzyme (Figure 11).

**Figure 12:**
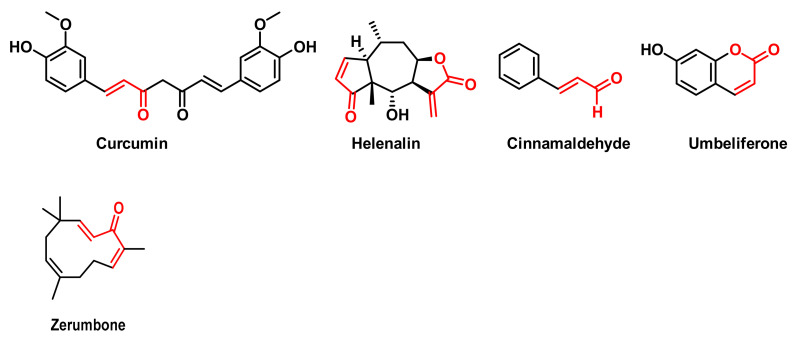
Structures of Curcumin, Helenalin, Cinnamaldehyde, Umbelliferone and Zerumbone.

As natural compounds we have selected known products that are MAs (Figure 12):

These natural compounds are also known for their remarkable anti-inflammatory, antioxidant and anticancer properties. These compounds have been shown to reduce inflammation by inhibiting pro-inflammatory cytokines and enzymes [114], help neutralise free radicals—thereby reducing OS and protecting cells—demonstrate the ability to induce apoptosis (programmed cell death) in various cancer cell lines, inhibit cancer cell proliferation and affect multiple cell signalling pathways involved in inflammation and cancer progression [115,116]. Curcumin, a polyphenolic compound derived from the rhizomes of the turmeric plant, *Curcuma longa*, is known for its anti-inflammatory, antioxidant and anti-tumour properties [117]. Helenalin is a sesquiterpene lactone found mainly in plants of the genus *Arnica*, such as *Arnica montana* and *Arnica chamissonis foliosa*, which has gained considerable attention due to its potent anti-inflammatory and anti-tumour properties [118]. Cinnamaldehyde, which gives cinnamon its characteristic aroma and flavour, has potential biological activities through inhibition of protein tyrosine phosphatase 1B (PTP1B), which may have implications for type 2 diabetes [119] and reduced viability of breast cancer cells [120]. Zerumbone is a cyclic sesquiterpene compound found in ginger (*Zingiber officinale*) and is especially abundant in *Zingiber zerumbet*. Its main properties include anti-inflammatory, antioxidant, anti-carcinogenic and anti-allergic action [121].

## 4. Structures and Location of Cys Residues

Cys residues are crucial for redox-dependent regulation, involved in a variety of cellular processes, so alkylation can significantly alter the function of these key proteins [122].

Cys497 and Sec498 are located at the C-terminal end of TrxR. Cys497 is located near helix 3, while Sec498 is close to helix 1 of the enzyme structure, creating a catalytic site where Trx is reduced [123]. Within the TrxR structure, the amino acids Cys497 and Sec498 form a unique redox centre. This C-terminal redox centre (Cys497/Sec498) is placed on the surface of the enzyme, allowing it to interact with its substrates, such as thioredoxin [124]

Cys528 in XPO1 is situated within the hydrophobic cargo-binding pocket of the protein. This pocket is part of the functional domain that interacts with nuclear export signals (NESs) and is essential for XPO1′s function as a nuclear exportin [125]. Cys528 is a reactive Cys residue, so it can form covalent bonds with electrophilic compounds and is located in a pocket within the XPO1 protein that is accessible to small molecules.

Cys259 in STAT3 is found in the coiled-coil domain of the protein. STAT3 is composed of six functional domains, and the coiled-coil domain is a critical region for STAT3 recruitment [126]. Cys259 can undergo PTMs and inhibit STAT3 activation by interfering with its phosphorylation and subsequent dimerization and nuclear translocation [127].

Cys38 is positioned in the p65 subunit of NF-κB and plays a crucial role in the regulation of NF-κB activity. Cys38 is an S-nitrosylation site on p65. When this PTM occurs, a decrease in the DNA binding capacity of p65 and an inhibition of its transcriptional activity are observed [128]. Alkylation of Cys38 disrupts DNA binding and inhibits NF-κB activity [76], since the p65 molecule altered by alkylation would no longer possess the reported binding specificity. The accessibility of Cys38 allows NF-κB to act as a sensor of cellular oxidative status, which is central to its role in regulating immune and inflammatory responses [129].

Cys179 is set within the IKKb activation loop, a critical region that regulates the enzyme’s activity. Cys179 plays a crucial role in facilitating the phosphorylation of serine residues Ser-177 and Ser-181 within the activation loop and essentially helps to create a conformation that allows the upstream kinases (like TAK1) to efficiently phosphorylate these serines, leading to IKKb activation [130]. Cys179 is a target for certain IKKb inhibitors, which act by modifying or interacting directly with it, thus interrupting the activation process.

Cys255 is located in the internal cavity of the PasB domain of HIF-1α protein, accessible to small molecules [93]. Cys255 is one of three Cys residues (with Cys337 and Cys334) present in areas potentially critical for HIF-1α/ARNT protein−protein interaction. It is a reactive site for the development of covalent inhibitors that can modulate the interaction between HIF-1α and ARNT (Aryl Hydrocarbon Receptor Nuclear Translocator) [93]. The key Cys or Sec residues that can be alkylated by MAs in enzymes and transcription factors often overexpressed in neoplastic cells are summarized in Table 1.

## 5. Local Docking of Michael Acceptor Compounds on Reactive Cysteines

MAs, thanks to their α,β-unsaturated groups, are highly reactive with biological nucleophiles such as the thiol group of Cys. This alkylation is a PTM that involves the addition of an alkyl group to the thiol group of a Cys residue. This modification can alter the structure and function of proteins, including TFs, which are proteins that regulate gene expression, thereby triggering or inhibiting pathological responses [131].

Covalent binding-based drugs offer certain advantages over classical non-covalent drugs [132], such as being able to use a lower dose as well as reducing the frequency of treatment, potentially reducing side effects [133]. Secondly, since the targets are cysteines present in proteins, shallow binding pockets can be used as targets [134]. Third, covalent drugs can bind to protein variants when non-covalent drugs fail [135].

The Michael reaction to form a specific covalent bond between a Cys of a protein and an electrophilic ligand occurs in two steps:

The first step is the approximation driven by electrostatic and van der Waals interactions. This initial step involves the approach of the electrophilic ligand to the active site of the protein where the reactive Cys is located. Non-covalent interactions, such as electrostatic and van der Waals forces, play a crucial role in this approach and orientation process. These forces play a crucial role in directing the ligand to the active site and orienting it correctly for the potential reaction. Electrostatic interactions involve attractions between opposite charges or dipoles and help in long-range recognition and initial positioning [136]. Van der Waals interactions are weak, short-range forces between molecules and include dipole−dipole interactions, induced dipole interactions and dispersion forces, and these interactions contribute to the fine-tuning of ligand positioning [137]. This step facilitates the correct alignment of the ligand and ensures that the electrophilic part is positioned close to the thiol group of the reactive cysteine, increasing the likelihood of the reaction occurring, and contributes to drug specificity. These interactions are specific for the ligand−protein pair, increasing selectivity.

The second step is the chemical reaction by electron exchange. Once the ligand is correctly positioned, the chemical reaction takes place. In this step, the thiol (SH) group of the Cys acts as a nucleophile, attacking the β-carbon of the α,β-unsaturated system of the electrophilic ligand. This nucleophilic attack results in the formation of a new covalent bond [138].

The approach driven by electrostatic and van der Waals interactions is often reversible because these interactions are generally weaker compared to covalent bonds, allowing for reversibility.

Over the last two decades, significant advances have been made in computational resources and methods in medicinal chemistry, with the aim of understanding more deeply the interactions between ligands and proteins [139]. Thus, their studies and evaluations have become easier, faster and cheaper thanks to computer-aided drug design tools, which can include molecular docking algorithms capable of studying covalent bonds and representing their formation, such as AutoDock (AD), Glider (CovDock) and others [140], even with covalent docking options, such as CovalentDock (built on AD) [141].

To provide additional support for our review article, we conducted a molecular docking study using AutoDockFR (ADFR) [142] to model and score the Michael reaction between cysteine residues in transcription factors and both anticancer drugs and natural products. ADFR software suit version 1.0 rcl for Linux is a software suite designed for docking calculations, based on AutoDock [143] and specialized in handling docking calculations with flexible residues. For this purpose, ADFR employs a genetic algorithm to explore and optimize both the ligand conformations and the poses of flexible residues. Additionally, it utilizes a scoring function based on gradient affinity maps and leverages the Dunbrack rotamer library [144] to select privileged rotamers of residues. ADFR incorporates a method to perform covalent docking that involves overlapping the target residue with a modified version of the ligand, in which a side chain containing the residue’s atoms is added, followed by selective flexible docking with the modified residue.

For this study, the RDKit Python package (version 2024.03.6) was used to modify the ligands by removing the double bond and introducing a CCCS chain at the Michael acceptor sites. Since this modification can generate one or two new chiral centers, all possible stereoisomers were considered. In addition, for molecules such as Helenalin, which contain multiple Michael acceptor sites, all possible regio-isomers were also generated. The ADFR suite’s prepare ligand script was used to prepare the modified structures for the docking studies.

Receptor structures were downloaded from the Protein Data Bank (PDB): 2ZZ0 for TrxR [145], 5DIS for XPO1 [146], 6NUQ for STAT3 [147], 1NFI for NF-κB [148], 4E3C for IKKβ and 4H6J for HIF-1 [93].

ChimeraX [149] was used to preprocess the structure by removing solvents, ligands and other protein chains, retaining only the transcription factors. The ADFR suite scripts, reduce [150], prepare receptor and AGFR [151], were used to add hydrogens, prepare the receptor and calculate the gradient affinity map, respectively. The search box was centred on the sulfur atom of the active cysteine, with a padding of 50 Å. Docking experiments were performed for each isomer−receptor pair. In addition to the target cysteine of each receptor, other residues were also set as flexible to improve the accuracy of the docking scores: Glu 297, Lys 537, Lys 568 and Glu 571 for XPO1; Gln 247, Glu 324, Arg 325 and Arg 350 for STAT3; Lys 37, Glu 39 and Arg 41 for NF-kB; Gln 48, Asn 54 and Glu 181 for IKKβ; Glu 168, Tyr 254, Leu 271 and Glu 268 for HIF1. In the case of TrxR, two separate covalent docking studies were conducted around either Cys 497 or Sec 498, setting the other residues as flexible along with Leu 409.

The results of the docking studies are summarized in Table 2, showing the AutoDock affinity scores expressed in kcal/mol. The lower score indicates a more energetically favorable binding. A negative score usually corresponds to enhanced contacts between the ligand and the receptor, forming a covalent binding with additional bond interactions (such as hydrogen bonds). These contacts often play a significant role in determining the strength and specificity of ligand−protein bonds. Table 2 presents the best score for the top isomer of each ligand−target combination.

In the assays with TrxR as the receptor, no significant preference was observed between Sec 498 and Cys 497 as the electrophilic site, except for Umbelliferone, whose addition to Cys 497 enables the formation of a hydrogen bond with Leu 493. However, covalent docking studies do not account for the differences in reactivity between selenium and sulfur; therefore, the higher electrophilicity of the selenol moiety may favor nucleophilic attack at Sec 498 [152].

**Figure 13:**
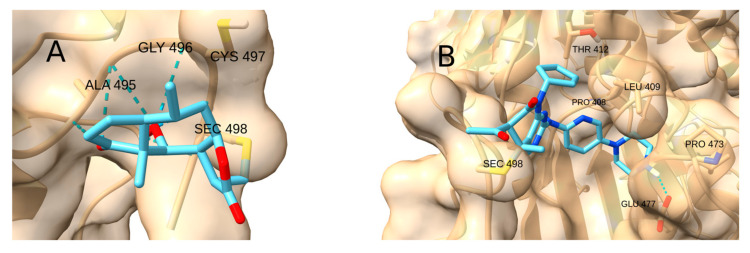
Two different interactions in the complexes with TrxR. (**A**) Helenalin forming hydrogen bonds with the backbone NH moieties. (**B**) Palbociclib inside the pocket.

Two different types of interactions were identified in the complexes with TrxR (Figure 13). The natural products Cinnamaldehyde, Curcumin, Helenalin, Zerumbone and Umbelliferone adsorb onto a surface formed by the backbone of residues 493–496, where the backbone NH moieties can form hydrogen bonds with the carbonyl group of the Michael acceptor system and other H bond acceptor groups. In contrast, the drugs CDDO-Me, Palbociclib, STAT3-IN-1 and Sunitinib fit into a pocket formed by residues 408–412 and 472–478. The highest affinity was observed for Palbociclib (−5.4 kcal/mol), whose polycyclic structure fits smoothly into that pocket and forms an H bond with Glu 477.

The active site of XPO1 contains Lys 537 and Lys 568, which can form hydrogen bonds with the carbonyl group of the Michael acceptor system, as well as with other hydrogen bond acceptors. In addition, near the reactive cysteine, there is a long hydrophobic pocket formed by residues Leu 525, Ala 541, Ile 544 and Met 545.

**Figure 14:**
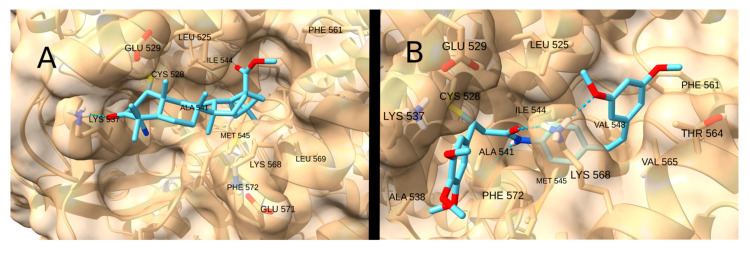
Interaction of CDDO-Me (**A**) and STAT3-IN-1 (**B**) with XPO1.

Consequently, the highest docking score was achieved by the terpenoid-like compound CDDO-Me (−9.3 kcal/mol; Figure 14A), which forms a hydrogen bond with LYS 537 and whose apolar regions fit into the hydrophobic pocket. Strong affinities were also observed for the drugs STAT3-IN-1 (−8.7 kcal/mol; Figure 14B), Palbociclib (−7.5 kcal/mol) and Sunitinib (−7.2 kcal/mol), as well as for the natural product curcumin (−7.6 kcal/mol), due to their ability to form two or more hydrogen bonds with the receptor residues.

**Figure 15:**
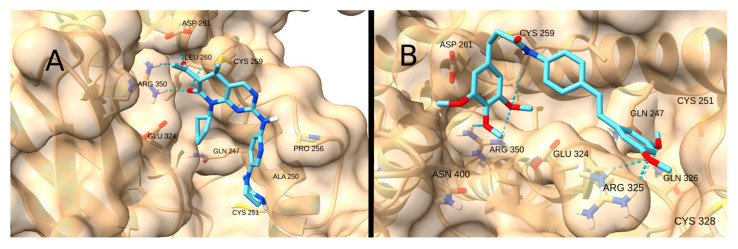
Interaction of Palbociclib (**A**) and STAT3-IN-1 (**B**) with STAT3.

Similarly, the STAT3 receptor site contains Arg 325 and Arg 350, which can also form hydrogen bonds with acceptors such as the carbonyl moiety of the Michael acceptor system. The highest scores were obtained for Palbociclib (−6.6 kcal/mol; Figure 15A) and STAT3-IN-1 (−6.0 kcal/mol; Figure 15B), which have multiple spatially separated acceptor groups, allowing them to interact with both Arg.

**Figure 16:**
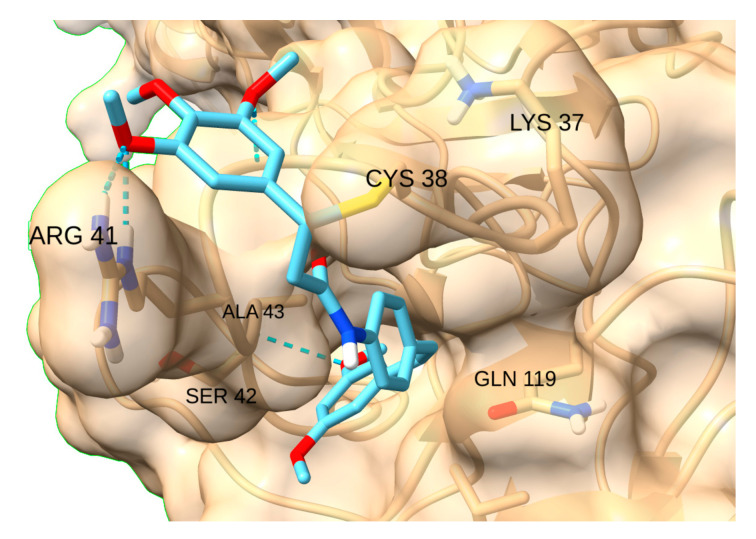
Interaction of STAT3-IN-1with NF-kB.

NF-kB has two hydrogen bond donors -Arg 41 and Lys 37- and a pocket intricately located near the reactive residue. STAT3-IN-1 was by far the most affine ligand (−7.7 kcal/mol), as its flexible structure allows the ligand to reach the pocket while simultaneously forming a hydrogen bond with Arg 41 (Figure 16).

**Figure 17:**
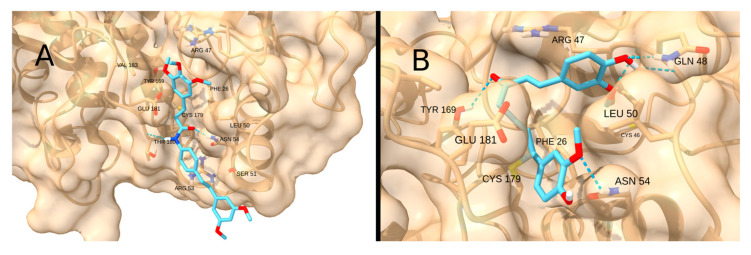
Interaction of STAT3-IN-1 (**A**) and Curcumin (**B**) with IKKß.

Unlike the previous receptors, the reactive site of IKKβ is highly hydrophilic due to the presence of polar residues in the pocket. Therefore, the interaction with the terpenoid-like molecule CDDO-Me is significantly restricted. The highest scores were obtained for the drug STAT3-IN-1 (−5.4 kcal/mol; Figure 17A) and the natural product curcumin (−5.4 kcal/mol; Figure 17B), both of which can form multiple hydrogen bonds with the polar residues in the pocket.

**Figure 18:**
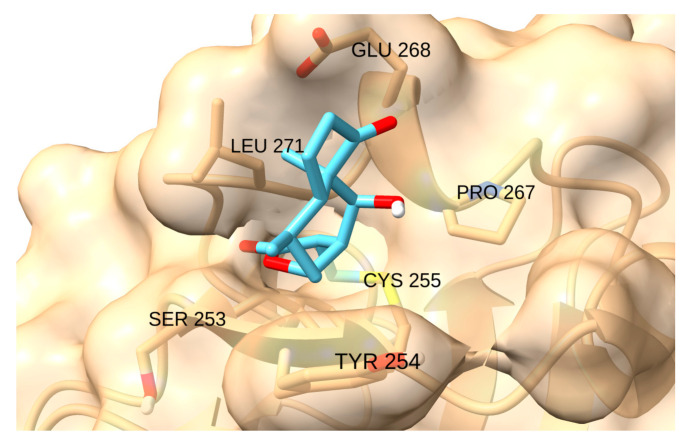
Interaction of helenalin with HIF-1.

Finally, HIF-1 has a small reactive pocket, which hinders the binding of large and rigid ligands. The interaction with CDDO-Me, Palbociclib, Sunitinib and Zerumbone is geometrically restricted. The natural product Helenalin showed the highest score (−4.4 kcal/mol), as the Michael addition to its terminal alkene moiety positions the bulk of the molecule away from the narrow cavity (Figure 18).

## 6. Conclusions

The review presented, together with the simulations performed, a molecular mechanism of inhibition of a key enzyme (TrxR) and several TFs by MA molecules, as well as a consideration of their possible influence on cancer cells. This study explored the therapeutic potential of targeting Cys residues in key proteins that are generally overexpressed in neoplastic cells. Through computational simulations, we demonstrated possible binding affinities between specific alkylating agents and these critical Cys sites. This suggests a plausible mechanism for covalent modification of TFs, thereby altering their function and potentially inhibiting tumorigenesis. Specifically, we focused on MAs, a class of electrophilic compounds, as potential agents for covalent modification of these Cys residues. Through computational simulations, we demonstrated strong binding affinities between MAs and critical Cys sites in enzymes and TFs. Simulations predict that some alkylating compounds could significantly disrupt protein−protein interactions, inhibiting the function of overexpressed proteins in cancer. This suggests a plausible mechanism for modulating TF activity and, consequently, inhibiting tumorigenesis.

Covalent inhibitors by Michael reaction are a powerful resource to target specific cysteines in enzymes and TFs, and computational applications of covalent docking are being developed to enhance the study of such compounds. In this study, we selected various TFs invoked and overexpressed in the neoplastic state based on the existing literature to evaluate the formation of covalent ligand−protein complexes, providing the basic stereochemistry prior to the Michael reaction. This analysis provides a basis for evaluating other covalent docking algorithms in the future.

Our docking assays showed that, for the majority of the receptors, the assayed anticancer drugs exhibited higher affinity due to their better fit in the receptor pockets. Among the natural products, curcumin, which is more similar to the drugs, also showed a higher docking score. The only exception was the HIF-1 receptor, where the best ligand was found to be the natural product helenalin, due to the favorable positioning of its terminal Michael acceptor system in the receptor’s confined binding site.

Future research should focus on validating these findings through in vitro and in vivo studies while exploring synergistic combinations of natural compounds and chemotherapeutics to maximize therapeutic efficacy against diverse cancer types. This includes in vitro and in vivo studies to assess the efficacy, selectivity and toxicity of Michael acceptor-based compounds. Additionally, optimizing the delivery of these compounds to cancer cells and minimizing off-target effects will be essential for developing safe and effective therapies.

## Figures and Tables

**Table 1:** Cysteines (Cys) or selenocysteines (Sec) residues of selected enzymes or transcription factors that can be alkylated by Michael acceptors.

Enzyme or Transcription Factor	Cys or Sec Residues
TrxR	Cys497 and Sec498
XPO1	Cys528
STAT3	Cys259
NF-kB	Cys38
IKKβ	Cys179
HIF-1	Cys255

**Table 2:** AutoDock scores for Curcumin, Cinnamaldehyde, Zerumbone, Helenalin, Umbelliferone, STAT3-IN-1, CDDO-Me, Sunitinib and Palbociclib versus TrxR, XPO1, STAT3, NF-kB, IKKβ and HIF-1. Covalent docking was performed using AutoDockFR software suit version 1.0 rcl for Linux. Positive values indicate an increase in the Gibbs free energy, which signifies that the addition reaction is not favorable and may not occur.

	TRXR Sec 498	TRXR Cys 497	XPO1	STAT3	NF-KB	IKKß	HIF-1
Curcumin	−3.8	−4.0	−7.6	−5.5	−6.0	−5.4	−2.9
Cinnamaldehyde	−1.1	−0.9	−3.6	−1.8	−3.1	−3.5	−2.5
Zerumbone	−2.1	−2.2	−5.5	−4.7	−4.3	−3.8	+5.0
Helenalin	−3.8	−3.0	−6.1	−4.4	−5.9	−4.4	−4.4
Umbelliferone	−0.5	−2.0	−5.0	−3.9	−3.4	−3.4	−1.2
Stat3-In-1	−4.4	−3.7	−8.7	−6.0	−7.7	−5.4	−1.6
Cddo-Me	−4.2	−4.4	−9.3	−5.1	−6.2	+2.0	+40.4
Sunitinib	−2.7	−2.8	−7.2	−4.0	−4.7	−4.4	+4.1
Palbociclib	−5.4	−4.4	−7.6	−6.6	−6.1	−2.0	+15.8
